# Effect of *ω*-3 Fatty Acid on Gastrointestinal Motility after Abdominal Operation in Rats

**DOI:** 10.1155/2011/152137

**Published:** 2011-03-16

**Authors:** Qun Zhang, Jian-Chun Yu, Wei-Ming Kang, Guang-Jin Zhu

**Affiliations:** ^1^Department of General Surgery, Peking Union Medical College Hospital, Peking Union Medical College, No. 1 Shuaifuyuan Wangfujing, Beijing 100730, China; ^2^Department of Pathophysiology, Peking Union Medical College, No. 1 Shuaifuyuan Wangfujing, Beijing 100730, China

## Abstract

*Objective*. To investigate whether *ω*-3 fatty acid could stimulate gastrointestinal motility after abdominal operation. *Method*. Wistar rats were randomly divided into 3 group (normal saline group, intralipid group, and *ω*-3 fatty acid group, *n* = 18/group) after partial caecectomy and gastrostomosis, each group was divided into 3 groups (POD1, POD3, and POD6, *n* = 6/group). Serum gastrin (GAS), motilin (MTL), interleukin-1 (IL-1), interleukin-6 (IL-6), tissue necrosis factor-*α* (TNF-*α*), cyclooxygenase-2 (COX-2), gastric emptying rate, and small bowel propulsion rate were measured. *Results*. On POD 3, gastric emptying rate and small bowel propulsion rate in *ω*-3 fatty acid group were higher than those in normal saline group and intralipid group. Serum GAS and MTL levels in *ω*-3 fatty acid group were higher than those in normal saline group, but serum IL-1, IL-6, TNF-*α*, and COX-2 levels were lower than those in normal saline group and intralipid group. *Conclusion*. *ω*-3 fatty acid could accelerate the recovery of gastrointestinal mobility after abdominal operation in rats, mainly by relieving postoperative inflammation.

## 1. Introduction

Early postoperative gastrointestinal motility dysfunction is a frequent complication of laparotomy, especially after gastrointestinal operation. It may cause small bowel obstruction, serious infection, hepatic dysfunction, or reoperation with prolonged hospitalization and increased perioperative expenses. In patients with treatment, continuous parenteral nutrition or enteral feeding is necessary, but the outcomes of such approaches are variable. 

During parenteral nutrition or enteral nutrition, numerous hormones secreted by the gut influence gastrointestinal motor, and appear to contribute to the pathogenesis of gastrointestinal motility dysfunction [1]. 

It is well established that manipulation of the bowel induces the secretion of proinflammatory cytokines, including interleukin-1 (IL-1), interleukin-6 (IL-6), and tissue necrosis factor-*α* (TNF-*α*), and then to impaired muscle function [2]. And cyclooxygenase-2 (COX-2) is the key enzyme of prostaglandin synthesis in inflamed tissues. It can affect the motor activity of gastrointestinal tract in postoperative ileus, and COX-2 inhibitors could help to improve the outcome of this gut disorder [3, 4].

Supplementation of enteral nutritional formulas and parenteral nutrition lipid emulsions with *ω*-3 fatty acid could help reduce inflammation [5, 6], but the relationship of *ω*-3 fatty acid and gastrointestinal motility still remains unknown.

Our research was designed to study whether *ω*-3 fatty acid could stimulate gastrointestinal motility after abdominal operation and its mechanisms of action.

## 2. Materials and Methods

54 Wistar rats with the average body weight 250 ± 20 g were randomly divided into normal saline group, intralipid group, and *ω*-3 fatty acid group (*n* = 18/group). Each group was divided into POD 1 group, POD 3 group, and POD 6 group (*n* = 6/group) according to the time of sacrifice. 

*Abdominal Operation*. Wistar rats were anaesthetized with 1% Pentobarbital (40 mg/K). Then, opened the abdominal cavity, partial caecectomy and gastrostomosis were performed. The gastric feeding tube was fixed on the back of rats. Therefore, it closed the abdominal cavity and waited for anaesthesia recovery.

The rats were perfused normal saline (12.5 mL/d), intralipid (12.5 mL/d, 5 g/kg*·*d), or *ω*-3 fatty acid (12.5 mL/d, 5 g/kg*·*d) through gastric feeding tube after operation.

On POD 1, POD 3, and POD 6, the rat gastric emptying rate, small bowel propulsion rate, serum gastrin (GAS), IL-1, IL-6, TNF-*α*, COX-2 were measured with ELISA methods (Uscnlife Science & Technoilgy Company, USA.). Serum motilin (MTL) was measured with RIA methods. MTL RIA kit (China institute of atomic energy, China) and Multigamma 1261 instrument (Finland) were used.

Phenolsulfonphthalein paste (0.1 mg/mL phenolsulfonphthalein, 10% gelatin) was injected into stomach through stomach-tube 25 min before rats were sacrificed. Gastric emptying rate was measured by gastric phenolsulfonphthalein emptying. Small bowel propulsion rate was measured by small bowel phenolsulfonphthalein propulsion rate.

 Continuous variables were compared using Student's *t*-test. *P* value less than .05 was considered significant.

## 3. Results

1. Gastric emptying rate, small bowel propulsion rate (see Tables 1 and 2).
2. Serum GAS and MTL levels (see Tables 3 and 4).
3. Serum IL-1, IL-6, TNF-*α* levels (see Tables 5, 6, 7 and 8).

On POD 3, gastric emptying rate, small bowel propulsion rate in *ω*-3 fatty acid group were higher than those in normal saline group and intralipid group. Serum IL-1, IL-6, TNF-*α*, COX-2 levels in *ω*-3 fatty acid group were lower than those in normal saline group and intralipid group. Serum GAS and MTL levels in *ω*-3 fatty acid group were higher than those in normal saline group.

## 4. Discussion

*ω*-3 fatty acid include Eicosapentaenoic acid (EPA), Ducosahexaenoic acid (DHA), and Alpha-linolenic acid (ALA), which play an important role in the regulation of inflammation [7].

The most important gastrointestinal hormones are GAS and MTL. GAS is secreted from neuroendocrine G cells which are principally located in the antrum of the stomach. MTL is a hormone released by the endocrine cells of the duodenal mucosa. They both can stimulate gastrointestinal motility.

Our research showed that on POD 3, the serum GAS and MTL levels in *ω*-3 fatty acid group were higher than those in normal saline group, but there was no difference between *ω*-3 fatty acid group and intralipid group. This indicates the mechanism that *ω*-3 fatty acid promotes the recovery of gastrointestinal motility does not lie in the increase of serum GAS and MTL levels. The possible reasons are as follows. (1) Different food has different effect on gastrointestinal hormones. Both *ω*-3 fatty acid and intralipid belong to fatty acids; their effects to stimulating gastrointestinal hormones' secretion are similar, but more powerful than normal saline. (2) *ω*-3 fatty acid and intralipid can inhibit the secretion of gastric acid, reduce PH value in stomach, then stimulate the secretion of GAS. (3) *ω*-3 fatty acid and intralipid can also stimulate the secretion of MTL.

As we know, intestinal handling induces the secretion of IL-1, IL-6 and TNF-*α*, triggers mast cell activation and inflammation associated with prolonged postoperative ileus. COX-2 catalyzes the first step of conversion of its common substrate, arachidonic acid (AA), into a number of biologically active derivatives, designated as prostaglandins (PGs, including PG-E2, PG-D2, PGF-2*α*, and PG-I2) and thromboxane (TX) A2, which are major participants in rodent postoperative ileus [8]. In our research, on POD 3, the serum IL-1, IL-6, TNF-*α*, COX-2 levels in *ω*-3 fatty acid group were lower than those in normal saline group and intralipid group, *P* < .05. This indicates that *ω*-3 fatty acid can decrease serum inflammatory factors and COX-2 levels. The possible reasons are as follows. (1) After consumption, *ω*-3 fatty acid can be incorporated into cell membranes and can reduce the amount of AA available for the synthesis of proinflammatory eicosanoids (e.g., PGs, leukotrienes (LTs)). Likewise, *ω*-3 fatty acid can also reduce the production of inflammatory cytokines, such as IL-1, IL-6, and TNF-*α* [9]. (2) With regard to inflammatory processes, the main fatty acids of interest are the AA, which is the precursor of inflammatory eicosanoids like PG-E2 and LT-B4, but *ω*-3 fatty acid gives rise to mediators (PG-E3, LT-B5) that are less inflammatory than those produced from AA [10]. (3) In addition to modifying the lipid mediator profile, *ω*-3 fatty acid exerts effects on other aspects of inflammation like leukocyte chemotaxis and inflammatory cytokine production. Some of these effects are likely due to changes in gene expression, as a result of altered transcription factor activity [10]. (4) COX-2 is the key enzyme of prostaglandin synthesis and *ω*-3 fatty acid can reduce the expression of COX-2 mRNA, in this respect, *ω*-3 fatty acid could inhibit inflammation [11, 12]. (5) Recently, resolvins and protectins, novel *ω*-3 fatty-acids-derived mediators were identified [13]. These mediators can signal for potent counter-regulatory effects on leukocyte functions, including preventing uncontrolled neutrophil swarming, decreasing the generation of cytokines, chemokines, and reactive oxygen species and promoting clearance of apoptotic neutrophils from inflamed tissues [14]. In this way, *ω*-3 fatty acids could reduce gastrointestinal inflammation. 

Because postoperative inflammation inhibits gastrointestinal mobility significantly, *ω*-3 fatty acid could accelerate the recovery of gastrointestinal mobility after abdominal operation.

Our study showed that enteral nutrition with *ω*-3 fatty acid compared to intralipid could accelerate the recovery of gastrointestinal mobility after caecectomy in rats. The mechanism of action is that *ω*-3 fatty acid could decrease serum inflammatory facts levels and relieve postoperative inflammation. Hence, it can accelerate the recovery of gastrointestinal mobility.

## 5. Conclusion

*ω*-3 fatty acid could accelerate the recovery of gastrointestinal mobility after abdominal operation in rats, mainly by decreasing serum levels of IL-1, IL-6, TNF-*α*, and COX-2 and by relieving postoperative inflammation.

## Figures and Tables

**Table 1:** Gastric emptying rate (%).

	POD1	POD3	POD6
(1) normal saline group (*n* = 6)	24.70 ± 2.73	34.82 ± 3.02	46.48 ± 3.25
(2) intralipid group (*n* = 6)	24.58 ± 2.54	37.32 ± 2.01	47.90 ± 2.21
(3) *ω*-3 fatty acid group (*n* = 6)	24.62 ± 2.32	40.35 ± 1.9	347.33 ± 4.03
*P* (between group 1 and 3)	0.959	0.004*	0.740
*P* (between group 2 and 3)	0.974	0.044*	0.820
*P* (between group 1 and 2)	0.935	0.095	0.401

**Table 2:** Small bowel propulsion rate (%).

	POD1	POD3	POD6
(1) Normal saline group (*n* = 6)	35.27 ± 3.09	40.48 ± 2.71	53.93 ± 2.49
(2) Intralipid group (*n* = 6)	35.35 ± 3.76	43.12 ± 2.34	55.32 ± 3.23
(3) *ω*-3 fatty acid group (*n* = 6)	35.33 ± 2.87	48.58 ± 3.18	56.80 ± 2.19
*P* (between group 1 and 3)	0.972	0.002*	0.164
*P* (between group 2 and 3)	0.992	0.008*	0.458
*P* (between group 1 and 2)	0.975	0.147	0.935

**Table 3:** Serum GAS (pg/mL).

	POD1	POD3	POD6
(1) normal saline group (*n* = 6)	93.78 ± 19.21	122.25 ± 18.78	153.58 ± 26.74
(2) intralipid group (*n* = 6)	92.00 ± 12.13	150.75 ± 20.301	51.55 ± 26.27
(3) *ω*-3 fatty acid group (*n* = 6)	94.67 ± 17.46	156.68 ± 22.10	154.23 ± 20.93
*P* (between group 1 and 3)	0.849	0.002*	0.968
*P* (between group 2 and 3)	0.517	0.287	0.870
*P* (between group 1 and 2)	0.574	0.001*	0.900

**Table 4:** MTL (pg/mL).

	POD1	POD3	POD6
(1) normal saline group (*n* = 6)	137.83 ± 17.34	185.25 ± 16.27	234.22 ± 24.68
(2) intralipid group (*n* = 6)	147.65 ± 15.29	227.62 ± 23.58	233.98 ± 24.31
(3) *ω*-3 fatty acid group (*n* = 6)	140.10 ± 20.48	225.45 ± 20.20	238.12 ± 24.81
*P* (between group 1 and 3)	0.881	0.002*	0.835
*P* (between group 2 and 3)	0.301	0.880	0.561
*P* (between group 1 and 2)	0.384	0.029*	0.989

**Table 5:** IL-1 (pg/mL).

	POD1	POD3	POD6
(1) normal saline group (*n* = 6)	75.92 ± 8.40	71.30 ± 4.76	54.45 ± 6.18
(2) intralipid group (*n* = 6)	77.15 ± 4.31	68.72 ± 4.15	53.45 ± 4.76
(3) *ω*-3 fatty acid group (*n* = 6)	74.80 ± 5.28	60.23 ± 5.51	54.28 ± 3.95
*P* (between group 1 and 3)	0.683	0.007*	0.941
*P* (between group 2 and 3)	0.366	0.004*	0.568
*P* (between group 1 and 2)	0.642	0.194	0.549

**Table 6:** IL-6 (pg/mL).

	POD1	POD3	POD6
(1) normal saline group (*n* = 6)	201.70 ± 22.58	217.97 ± 32.631	43.70 ± 30.79
(2) intralipid group (*n* = 6)	215.83 ± 32.48	216.63 ± 27.96	127.83 ± 14.08
(3) *ω*-3 fatty acid group (*n* = 6)	207.83 ± 16.62	173.98 ± 12.76	142.22 ± 20.64
*P* (between group 1 and 3)	0.545	0.015*	0.907
*P* (between group 2 and 3)	0.519	0.011*	0.216
*P* (between group 1 and 2)	0.325	0.672	0.347

**Table 7:** TNF-*α* (pg/mL).

	POD1	POD3	POD6
(1) normal saline group (*n* = 6)	15.03 ± 2.02	13.97 ± 1.86	9.75 ± 1.58
(2) intralipid group (*n* = 6)	15.57 ± 1.77	14.83 ± 1.65	10.70 ± 2.34
(3) *ω*-3 fatty acid group (*n* = 6)	14.00 ± 3.07	12.50 ± 1.70	9.87 ± 1.43
*P* (between group 1 and 3)	0.439	0.007*	0.871
*P* (between group 2 and 3)	0.251	0.015*	0.255
*P* (between group 1 and 2)	0.603	0.672	0.347

**Table 8:** COX-2 (ng/mL).

	POD1	POD3	POD6
(1) normal saline group (*n* = 6)	15.03 ± 2.02	13.97 ± 1.86	9.75 ± 1.58
(2) intralipid group (*n* = 6)	15.57 ± 1.77	14.83 ± 1.65	10.70 ± 2.34
(3) *ω*-3 fatty acid group (*n* = 6)	14.00 ± 3.07	12.50 ± 1.70	9.87 ± 1.43
*P* (between group 1 and 3)	0.439	0.007*	0.871
*P* (between group 2 and 3)	0.251	0.015*	0.255
*P* (between group 1 and 2)	0.603	0.672	0.347

## References

[1] Khoo J, Rayner CK, Feinle-Bisset C, Jones KL, Horowitz M (2010). Gastrointestinal hormonal dysfunction in gastroparesis and functional dyspepsia. *Neurogastroenterology and Motility*.

[2] Bauer AJ, Boeckxstaens GE (2004). Mechanisms of postoperative ileus. *Neurogastroenterology and Motility*.

[3] Fornai M, Antonioli L, Colucci R (2010). Emerging role of cyclooxygenase isoforms in the control of gastrointestinal neuromuscular functions. *Pharmacology and Therapeutics*.

[4] Zhang Q, Yu JC, Ma ZQ, Kang WM, Ke MY, Qian JM (2006). The effects of enteral nutrition vs parenteral nutrition on gastric motility and gastroenteric hormones after subtotal gastrectomy: a perspective randomized compared clinical trial. *Zhonghua Wai Ke Za Zhi*.

[5] Martin JM, Stapleton RD (2010). Omega-3 fatty acids in critical illness. *Nutrition Reviews*.

[6] Stewart D, Waxman K (2007). Management of postoperative ileus. *American Journal of Therapeutics*.

[7] Wall R, Ross RP, Fitzgerald GF, Stanton C (2010). Fatty acids from fish: the anti-inflammatory potential of long-chain omega-3 fatty acids. *Nutrition Reviews*.

[8] Schwarz NT, Kalff JC, Türler A (2001). Prostanoid production via COX-2 as a causative mechanism of rodent postoperative ileus. *Gastroenterology*.

[9] Fetterman JW, Zdanowicz MM (2009). Therapeutic potential of n-3 polyunsaturated fatty acids in disease. *American Journal of Health-System Pharmacy*.

[10] Kelley DS, Taylor PC, Nelson GJ (1999). Docosahexaenoic acid ingestion inhibits natural killer cell activity and production of inflammatory mediators in young healthy men. *Lipids*.

[11] Mund RC, Pizato N, Bonatto S (2007). Decreased tumor growth in Walker 256 tumor-bearing rats chronically supplemented with fish oil involves COX-2 and PGE2 reduction associated with apoptosis and increased peroxidation. *Prostaglandins Leukotrienes and Essential Fatty Acids*.

[12] Calder PC (2003). n-3 polyunsaturated fatty acids and inflammation: from molecular biology to the clinic. *Lipids*.

[13] Serhan CN, Arita M, Hong S, Gotlinger K (2004). Resolvins, docosatrienes, and neuroprotectins, novel omega-3-derived mediators, and their endogenous aspirin-triggered epimers. *Lipids*.

[14] Levy BD (2010). Resolvins and protectins: natural pharmacophores for resolution biology. *Prostaglandins Leukotrienes and Essential Fatty Acids*.

